# Childhood Obesity and Academic Outcomes in Young Adulthood

**DOI:** 10.3390/children5110150

**Published:** 2018-11-13

**Authors:** Igor Ryabov

**Affiliations:** Department of Sociology and Anthropology, The University of Texas Rio Grande Valley, 1201 W University Dr, Edinburg, TX 78539, USA; igor.ryabov@utrgv.edu; Tel.: +1-956-665-7874

**Keywords:** obesity, academic outcomes, race–ethnicity, immigrant generational status

## Abstract

The present study used nationally representative data from the National Longitudinal Study of Adolescent Health (a.k.a., Add Health) to examine the impact of childhood obesity on young adult educational attainment. In addition to weight status, independent variables included race–ethnicity, immigrant generational status, family socio-economic status (SES), preference for overweight and obese friends in school, school socio-economic and race–ethnic composition, and other important predictors. Educational attainment was measured as a categorical variable with the categories reflecting key educational benchmarks: (1) being a high school graduate; (2) having some college education; and (3) having completed a bachelor’s or higher degree. The results indicate that in general, individuals who were obese as children are less likely to transition from high school to college, and even less likely to obtain a baccalaureate or more advanced degree. In line with the social network hypothesis of the obesity epidemic, we also found that having overweight and obese friends drives down the odds of educational success. Attendance at a higher SES school or a school with a lower percentage of minority students was positively associated with the odds of college attendance and obtaining a baccalaureate. Other important effects included race–ethnicity and immigrant generational status.

## 1. Introduction

Childhood obesity has become a major public health concern in the United States, with its prevalence having tripled over the last three decades. It is estimated that approximately 17% of our nation’s children and adolescents are now obese [1,2]. Apart from being a major antecedent of adult obesity, childhood obesity has long-term consequences through the accumulation of various health risk factors over the life course [3,4,5]. Childhood obesity incurs not only health risks in adulthood, but also high societal costs [5,6]. The limited evidence suggests that the social burden of childhood obesity includes lasting effects on economic mobility [7,8]. Regrettably, only a handful of studies have directly questioned the general proposition that childhood obesity affects academic attainment in late adolescence and young adulthood [9,10,11]. 

The present study is intended to contribute to this strand of research and examine the impact of weight status in childhood on a number of educational outcomes in young adulthood. Investigating the roles of immigrant generational status, race–ethnicity, and friendship preferences for overweight and obese friends in shaping the relationship between childhood weight status and the academic outcomes of young adults is another objective of this research. Three educational outcomes are examined: the probabilities of graduating from high school, some college education (but no bachelor’s degree), and having a bachelor’s degree or higher. The key predictor is weight status in childhood measured as a categorical variable distinguishing the healthy weight, overweight, and obese. The independent variables also include individual (immigrant generational status, race–ethnicity, family socio-economic background effects, age, and gender) as well as school level (percentage of minority children and average school socio-economic status (SES)) correlates of educational outcomes. The empirical base of the study is the National Longitudinal Study of Adolescent Health (henceforward, the Add Health), which is a dataset that allowed for the in-depth exploration of the relative roles of the aforementioned predictors of educational outcomes. It is important to mention that the Add Health is a panel survey, and thus, it can address the causal order of the relationship between childhood weight status and educational attainment in adulthood, while controlling for the confounding effects of individual socio-demographic and school characteristics. 

## 2. Background

In adults, research clearly demonstrates the adverse socio-economic consequences of obesity [12,13]. Being overweight or obese exposes one to the risk of poverty and downward social mobility [14,15,16]. Moreover, overweight and obese adults tend to have lower educational attainment than their normal weight counterparts [12,17,18]. Influenced by the executive function hypothesis, a theoretical framework that links physical exercise in adults with performance on executive function tasks [19,20], several studies found evidence that excessive weight in adults inhibits their cognition [21,22,23]. These developments have given rise to a small but growing body of literature that explores the relationship between weight status and cognitive development in children [24,25]. So far, the dominant focus has been on cognitive development per se, rather than achievement, and on preschool and early school-age children, rather than on adolescents. Although the existing evidence tends to suggest that overweight and obese youth perform worse in school that their healthy-weight counterparts, the number of studies is limited, their individual power is low (i.e., small sample sizes), and they lack extensive controls [26]. Most importantly, this research is almost exclusively cross-national in nature, which means that a long-term impact of childhood obesity on adult educational attainment remains underexamined.

Only three studies attempted to model adult educational outcomes as a function of body weight in childhood and adolescence. Using the National Longitudinal Survey of Youth data, Fowler-Brown et al. [11] examined the relationship between adolescent obesity and college degree attainment. Two samples were analyzed: one (approximately 4000 persons) that included adults who were adolescents (aged 14–18) in 1997, and another one comprised over 3000 individuals who were adolescents (aged 16–18) in 1979. The authors found mixed results. In the older cohort (adolescents in 1979), there were no differences in college degree attainment by adolescent weight. However, in the recent cohort (adolescents in 1997), individuals who were a normal weight as adolescents had a higher prevalence of college degree attainment compared to obese adolescents. Using the same dataset, Crosnoe [9] tested a social psychological model of the gendered link between obesity and education. The author reported that in schools where the average body mass index (BMI) of the student body was lower than the average obese woman were half as likely to attend college than healthy-weight women. However, in schools where female obesity was more prevalent, obese women had the same chance of attending college as non-obese women. A similar study by Crosnoe and Muller [10] documented that the relationship between obesity and lower academic achievement was stronger in schools with a lower average body size among students. Although the aforementioned studies examine different academic outcomes, certain commonalities are evident: (1) examining a single educational outcome, thus ignoring the continuous nature of the educational process; (2) taking a narrow view of the school context as the prevalence of obesity in a school; and (3) focusing on a limited number of explanatory variables, thus ignoring factors that can attenuate the relationship between an individual’s weight status in adolescence and academic outcomes in young adulthood.

In an attempt to underscore school context in determining an individual’s weight status, Crosnoe [9] and Crosnoe and Muller [10] used the average BMI of a school to capture the prevalence of obesity as a norm in the educational setting. In the present study, we approach the concept of the school context differently. We argue that the distinction that should be made is between school context per se and peer friendships in the school context. Apart from being educational institutions, schools are an important venue for the formation of adolescent friendship networks with specific values and codes of behavior [27,28]. In other words, schools are sites of social capital formation [29,30,31,32]. 

The importance of social networks is highlighted in both the literature stemming from social capital theory [27,28,29,30,31,32,33] and that which relates to social network hypothesis of obesity epidemic [17,34,35,36]. The former literature suggests that obesity is a social disease, and social networks facilitate its epidemic [34]. Empirical studies testing this hypothesis found that in different age–sex population groups (men and women, adults and adolescents alike), obesity spreads through people’s social networks [31,36]. Peers can become influential to individual’s behaviors, particularly when conformity helps individuals gain social status among their peers. Having obese peers may alter a person’s views on obesity and impact health-related behaviors such as diet and exercise [17,34,36].

One practical caveat is that it is essential to maintain the conceptual and empirical clarity when treating: (1) the composition of social setting (i.e., average BMI of a school) that provides an opportunity for the formation of social ties, and (2) the composition of social networks that are formed and operate in this social setting (i.e., BMI of a friendship network). Individuals are expected to have friendship networks that reflect school composition. However, the compositional effect on peer networks can be offset by a behavioral influence on patterns of friendship formation [28,29,30]. Put differently, adolescents tend to form friendships with similar others, thus displaying behavior known as homophily [29,30,32]. Therefore, overweight students are likely to choose one another as friends and become connected, regardless of the average weight status of the school they attend. In practical terms, it means that the average BMI of a school may not be the perfect approximation of the influence of the school context on individual weight-related behaviors. 

The present investigation adds to the literature by using longitudinal data from Add Health to examine the effects of adolescent obesity on young adult educational outcomes. Our study bridges several literatures (racial disparities, migration studies, social networks) to explore different scenarios that could potentially cause spurious associations between adolescent weight and later educational attainment. Specifically, we investigate whether and to what extent the relationship between weight status in adolescence and academic attainment in young adulthood is explained by the following factors: (1) race/ethnicity; (2) immigrant generations status; or (3) peer networks. Concerning the former, this study is unique in considering both: (1) a school’s student body composition that provides an opportunity for the formation of social ties, and (2) the actual composition of a school’s social networks.

## 3. Materials and Methods

### 3.1. Data

Data came from the National Longitudinal Study of Adolescent Health (hereafter, Add Health), which is a national longitudinal survey of adolescents and young adults. The survey was conducted by the Carolina Population Center at the University of North Carolina at Chapel Hill. The first round of Add Health data was collected in 1995. Since then, four follow-ups have been conducted: Wave 1 in 1994–1995, Wave 2 in 1996, Wave 3 in 2001–2002, and the last (Wave 4) in 2007–2008, when respondents were aged between 24–32. The Add Health used a school-based stratified sample design (the survey methodology is described in detail by Bearman et al. [37]). A systematic random sample of high schools along with feeder schools (i.e., middle schools whose students matriculate at the selected high school) was selected. Overall, 79% of the schools that were contacted agreed to participate in the study. All of the participating schools were stratified by sex and grade, with students randomly chosen within each stratum. During Wave 1, all of the students in the participating schools were surveyed (N = 90,118). A subset of students (N = 20,745) was randomly selected for in-home interviews, constituting Wave 1 data. Approximately one year after the in-school survey, Add Health administrators interviewed these adolescents in their homes. 

The in-home interviews were collected in all four waves. In the present study, all of the information on independent variables was obtained from Wave 1 in-home interview data, while the outcome variables were measured at Wave 4 (also in-home interview data). Wave 4 interviews were completed with 15,701 of the Add Health respondents (aged 24–32; 77.4% and 80.3% of eligible respondents, respectively). Those cases with missing values on anthropometric measurements (weight and height) used to compute the weight status, as well as at least one educational outcome of the interest, were excluded (N = 3610). Applying this selection criterion reduced the final sample size to 12,091 students from 129 schools. Missing values for all of the independent variables, except the weight status, were handled using multiple imputation methods, including the Markov chain Monte Carlo technique, with the efficiency of the resulting estimates within a 95% confidence interval (for more information see Rubin [38,39]). This procedure entails iteratively replacing missing values with predictions based on the variance–covariance matrix of the study variables, and assuming that the data are missing at random. Data are imputed using the Stata 10.0 software. Empirical results are averaged across the five imputation samples [40,41]. It is important to mention that the research ethics board review is not required for the present study, because it relies exclusively on a secondary use of anonymous information. This information was originally collected for a purpose other than the current research purpose, and was obtained from the sources open to the general public. The presentation or dissemination of results will not generate identifiable information.

### 3.2. Measures

Our primary interest is in understanding how weight status in childhood affects the academic attainment of young adults after most of their school-to-work transitions have been completed. Three educational outcomes were measured as the odds of: (1) being a high school graduate; (2) having some college education; or (2) holding a bachelor’s degree or higher (as of Wave 4 of the Add Health). Currently, the most widely used indicator of weight status is the body mass index (BMI) [42,43]. The BMI is calculated by dividing weight in kilograms by height squared in meters. To estimate weight status, we used the standard endorsed by the Centers for Disease Control and Prevention for overweight using BMI cut-off points for age and sex (for more information, see Cole et al. [42]). Overweight and obesity were defined as having a BMI at or above the 85th and 95th percentiles, respectively, for age and gender. In Wave I, anthropometric measurements were obtained as self-reports of the respondents. Although it is entirely possible that some students did not know how their exact height and weight, there is no reason why self-reported weight and height measures should be differently biased between cases and controls. Interviewer measures of height and weight were included at Wave II, and they were found to be correlated (Pearson’s correlation coefficient = 0.95) with the adolescent self-reports for Wave II [44]). Moreover, previous research studies indicate a very high correlation between self-reported and measured BMI among young people [45].

Race–ethnicity, one of the focus variables, is represented by four dichotomous variables for African-American, Asian American, Hispanic, and non-Hispanic white. These categories are mutually exclusive, and non-Hispanic whites were the reference category for the purpose of this analysis. Another important variable that is known to affect weight status—immigrant generational status—is measured by three variables for first, second, and higher generation immigrants. Those born outside the United States were counted as first-generation immigrants, and the second generation included those with at least one foreign-born parent. The rest of the respondents were classified as third or higher-generation immigrants, which is the reference category in our analyses.

In order to avoid the spurious correlation between weight status and later educational attainment, this study controls for individual and school-level factors associated with both obesity and educational outcomes. Hence, the individual-level characteristics that were used as control variables included parents’ income and educational attainment (in years), being raised in a two-parent family, sex (reference: female), and age. For the exception of the latter predictor (age), all of the independent variables were obtained from the Add Health Wave 1 in-home survey. 

An important variable in this study is the obesity saliency index (OSI), which measures the likelihood of having a friendship with an overweight or obese student conditional on the share of overweight and obese students in the school. The index was calculated as follows:

(1) $$Obesity\;Saliency\;Index\;=\;\frac{Percentage\;of\;Friends\;Who\;Are\;Overweight/Obese}{Percentage\;of\;Overweight\;and\;Obese\;Students\;in\;School}$$

This measure was derived from friendship nominations reported by respondents at Wave 1. For each participating school, the Add Health obtained a roster of its students and assigned them identification numbers. These rosters enabled the students to identify five friends of each gender (and, by implication, their weight status). We use this information to calculate the OSI, which is the relative preference for overweight and obese friends conditionally based on the average weight status of peers in the school. First, we identified all of the overweight and obese students among the friends at the school nominated by the respondent. Then, we divided this number by the number of all of the friends identified by the respondent. In order to calculate the percentage of overweight and obese students in each study school, we identified all of the overweight and obese students in the school, and then divided this number by the total number of students in the school. In order to account for the status of the community where the school is located, two measures were entered in the analyses at the school level: percentage of minority students and average SES. The percentage of minority students was computed as the percentage of African-American and Hispanic students, and was aggregated from the person-level cases within each school. We did not count Asian Americans as minorities in this case, because prior research shows that African-American and Latinos attend schools with far higher minority student enrollment than whites, while Asian Americans attend the most integrated schools [46]. The school SES composite was constructed as the sum of the standardized scores of parent’s income and education across all of the person-level cases within each school. This is appropriate, as these two variables are strongly intercorrelated at the school level (Pearson’s r = 0.90), but not at the individual level (Pearson’s r = 0.67). The control variables included age and gender.

### 3.3. Analytic Strategy

Given the multilevel, hierarchical structure of the Add Health dataset and the dependent variables being dichotomous outcomes, multilevel logistic regression was used as an appropriate technique. Individuals were used as level-1 units, while schools were used as level-2 units. The multivariate analyses were performed using the STATA software. To account for the stratified and clustered nature of the Add Health data, we weighted all of the analyses and adjusted standard errors for school-level clustering. The regression coefficients were obtained via maximum likelihood estimation, since the ordinary least squares estimation is not suited for the estimation of the logistic distribution.

Our multivariate regression models consist of three sets of analyses (Tables 2–4) that were designed to predict three levels of educational attainment in young adulthood. Parallel analyses were estimated for all three educational outcomes. Model 1 documented the effects of weight status only. Models 2, 3, and 4 added, respectively, race/ethnicity, generational status, family effects, and other controls (age and gender). Model 5 added the individual-level measure of obesity saliency as a way of testing the hypothesis that the preference for overweight and obese friends has a negative impact on one’s educational attainment. The final two models incorporate school-level factors, such as the percentage of minority students, and school SES. 

## 4. Results

### 4.1. Descriptive Analysis

An overview of the descriptive statistics across weight status categories is provided in Table 1. Obese individuals constitute, correspondingly, the largest and the smallest shares of young adults in the lowest (i.e., high school or less) and the highest (bachelor’s degree or higher) educational categories. At the same time, 40% of bachelor’s degree holders and 45% of respondents with some college education are healthy-weight individuals. The cross-tabulation of race–ethnicity and weight status (rows 5–9 of Table 1) reveals that although non-Hispanic whites comprise the majority of respondents in all of the weight status categories, African-Americans and Latinos are overrepresented among the overweight and obese. African-Americans constitute 22% and 26% of overweight and obese individuals, respectively. In contrast, the representation of African-Americans among healthy-weight individuals is low: only 13%. Likewise, 16% and 19% of overweight and obese individuals, correspondingly, are Latinos, while the share of Latinos among healthy-weight individuals is 11%.

The majority of the Add Health respondents in all of the weight status categories were native-parentage adults (i.e., generation 3; see Row 13 of Table 1). Nevertheless, there are noticeable disparities in weight status between immigrant generation groups. This is especially the case with the first-generation group, which comprise only 2% of obese, but 7% of healthy-weight individuals. The distribution of the second-generation group across the weight status categories is less divergent than that of first generation. Generation 2 comprises approximately equal shares of overweight (10%) and healthy-weight (11%) respondents, but a lesser share of the obese (7%).

There are noticeable differences in parents’ income and education between weight status categories. The difference in the parental educational attainment between the obese and the healthy weight constitutes 1.8 years, while the overweight score is between the other two weight status categories on the level of this indicator. The distribution of parents’ income follows a parallel pattern across weight status categories. The respondents who were raised in a two-parent family constituted approximately 53%, 44%, and 60% of the overweight, obese, and healthy-weight individuals, respectively. The sample’s sex ratio was relatively balanced, with females slightly outnumbering males (by 1–4% across weight status categories). The average age of the Add Health respondents as of Wave 4 was approximately 28 years.

### 4.2. Multivariate Analyses

Table 2 shows multilevel logistic models predicting the odds of having the lowest level of educational attainment: high school diploma or less. The baseline model of Table 2 shows that if no other predictors are added, overweight and obese individuals are significantly more likely to remain high-school graduates in young adulthood (the average age of respondents is approximately 28 years). These weight status discrepancies remain robust, even after taking into account race–ethnicity and other important factors. In the full model (model 6), which controls for all individual and school-level factors, the odds of holding a high school diploma is 13% and 20% higher for overweight and obese respondents, correspondingly, than for the healthy-weight individuals. Adding race–ethnicity variables in model 2 does not significantly alter the weight status effects. African-American and Latinos are predicted to have higher chances of remaining high-school graduates by their late 20s than non-Hispanic whites. These race–ethnicity effects are robust to the inclusion of other controls in the subsequent models of Table 2. The effect for Asian Americans, albeit significant in model 2, loses its significance in model 3, which accounts for immigrant generational status. In model 3, the coefficients for first and second-generation respondents are significant. Both immigrant generations were less likely to be high school diploma holders than the third-generation respondents. However, the effect for the second-generation respondents became insignificant with the addition of overweight saliency in model 5. All of the family background effects that were added in model 4 are significant and in the directions predicted from the literature [47,48]. The odds of holding a high school diploma at Wave 4 of the Add Health are predicted to be significantly lower for respondents who grew up in two-parent families and in families with higher SES (proxies by parents’ income and educational attainment). The effect of overweight saliency, which is a measure that taps preference for overweight and obese friends conditional on the weight status composition of the school, is significant in models 5 and 6 of Table 2. As predicted, a preference for overweight and obese friends results in higher odds of being a high school graduate by the age of 28. The school-level effects are added in model 6 (full model). Both of them are significant, albeit in the opposite direction. Respondents who attended schools with higher proportions of minority students, but a lower average SES of the student body, tended to remain high school graduates by Wave 4 of the Add Health.

Table 3 shows the results from models predicting the odds of having some college education. In the baseline model, we see some evidence of a reversal of the weight status disparity observed in Table 2. The odds of having some college education are lower for the obese than for the healthy weight in all of the Table 3 models. Put differently, compared to the healthy-weight individuals, obese respondents are less likely to obtain any college education approximately by the age of 28. It is important to know that this result is robust to the inclusion of race–ethnicity, socio-economic background, friendship preferences (obesity saliency), school-level variables, and other controls. The effect for overweight is in the same direction as in Table 2. It shows that the overweight are more likely to have some college education than their healthy-weight peers. It is worth noting that this effect is significant only in the baseline model. Thus, after the addition of race–ethnicity measures in model 2 of Table 3, the effect for overweight becomes insignificant. It is also insignificant in all of the other models of Table 3 that follow. Thus, after controlling for race–ethnicity and other important predictors of educational attainment, the odds of having some college education do not differ significantly between the overweight and healthy-weight groups. Two ethnic origin effects are consistently significant in all of the Table 3 models.

In line with prior research [49,50], we find that African Americans and Latinos are less likely to have some college education than non-Hispanic whites (the reference category). An inspection of immigrant generation effects reveals that the disparities in the odds of some college education between immigrants (first generation) and native-born respondents of native-parentage (third generation) are significant, but between children of immigrants (second generation) and the third generation are not. In the full model, the odds of having some college education for the first generation are approximately 15% higher than for the third generation. Except for family structure (being raised in a two-parent family is the reference category), all of the family effects in Table 3 are significant and in directions that are consistent with findings from prior research [51,52,53].

A negative association is found between some college education, on the one hand, and parents’ income and education, on the other. None of the other individual-level effects (age, gender, obesity saliency) reached significance at the *p* < 0.05 level. Only one school-level effect is significant (at *p* < 0.05), that of average SES. Hence, those who attended schools with a higher SES are more likely to attend college by the age of 28. The result is fully consistent with prior research [50,53,54].

In Table 4, we repeat the above analyses, with the difference being that the outcome variable is the odds of holding a baccalaureate or higher degree. An examination of model 1 of Table 4 reveals that compared to the healthy-weight individuals, overweight and obese individuals are less likely to hold a bachelor’s degree or higher. Similar to Table 3, Asian Americans and Latinos appear to be at a disadvantage vis-a-vis non-Hispanic whites in terms of college education. In all of the regression models of Table 4, these two minority groups are less likely to hold a baccalaureate or higher degree than non-Hispanic whites. The effect for Asian Americans is not consistently significant across Table 4 models. Asian Americans tend to have some advantage over non-Hispanic whites in models 2–4 of Table 4, but with the addition of overweight saliency, the Asian-American effect becomes insignificant. Immigrants tend to consistently outperform the native-parentage adults (third generation) on the likelihood of getting a baccalaureate or higher degree. Nevertheless, the effect for children of immigrants (second generation) is inconsistent. After controlling for all of the factors related to academic attainment, the probability of obtaining a college degree appears to be about the same for the second and third generations. As in the preceding Table 3 that predicts the odds of college education (degree), all of the family background effects are significant and in a predicted direction. The higher their parents’ income and educational attainment, the more likely a person is to obtain a college degree. Likewise, being raised in a two-parent family increases one’s odds of completing college with a bachelor’s or higher degree. Similarly to the preceding tables 2 and 3, neither age nor gender are significant in the full model of Table 4. The effects of overweight saliency and school effects that are entered in models 5 and 6, respectively, are all significant. Having overweight and obese friends (conditional on their availability) significantly lowers the odds of becoming a baccalaureate-holder by the age of 28. In line with our expectations, attending a school with a higher average SES but lower percentage of minority students significantly increases the odds of holding the baccalaureate.

## 5. Discussion

Considered together, the evidence presented in this article shows that obesity in and of itself drives down the odds of educational success. Together with socio-demographic predictors of educational attainment that have received abundant attention in the literature (e.g., race–ethnicity, immigrant generational status) and those that have not (e.g., socio-economic and race–ethnic composition of the school), weight status in childhood appears to have a profound effect on one’s chances of receiving tertiary education. Specifically, we found that the odds of having some college education and of holding a baccalaureate or more advanced degree are noticeably lower for respondents who were obese as children than for those who were of a healthy weight. The odds of having a bachelor’s degree are also significantly lower for those who were overweight as children. At the same time, the odds of remaining a high school graduate (or having received some schooling, but not finishing high school) in young adulthood are significantly higher for respondents falling into the top two weight status categories than for the healthy-weight category. All in all, a higher weight status in childhood is associated with lower academic attainment in young adulthood.

While the results for weight status are the most substantively important, other results are worth noting. First, in line with early studies [49,50], we find sizeable black–white and Latino–white gaps in attainment. Ceteris paribus, both African-Americans and Latinos are less likely to be college educated and be awarded a bachelor’s or higher degree. It is important to emphasize that these disparities were found while controlling for weight status and other effects on attainment. Second, as expected, we found that first-generation immigrants tend to outperform non-immigrants academically. However, we did not find significant differences in the odds of being a high school graduate, having some college education, or holding a bachelor’s degree between second and third generations, that is between children of immigrants and United States (US)-born respondents of native parentage. This finding is consistent with the prediction of the second-generation decline hypothesis [55] that purports that the second generation will be worse off academically and otherwise that their parents, who were first-generation immigrants. Third, our results indicate that having overweight or obese friends drives down the odds of educational success. This finding lends significant support to the social network hypothesis that suggests that social networks facilitate the spread of the obesity epidemic [34]. Finally, we also found that a school’s SES positively and significantly affects the educational attainment of all of the students, regardless of weight status and socio-economic background. At the same time, individuals who attended schools with higher concentrations of minority students tended to lag academically. These results are generally consistent with prior research [56,57]. 

This study has several limitations that must be acknowledged. First, the dependent variable, educational attainment, is represented by three discrete outcomes. Similarly, weight status, a categorical variable, was used to estimate the aforementioned outcomes. It is worth mentioning that classifying a heterogeneous population into mutually exclusive subgroups always involves some level of arbitrariness, and different criteria (and cut-off points) have been proposed to rationalize the selection of categories [42]. Perhaps, if continuous measures had been considered (i.e., educational attainment in years, BMI), the results might have been different. Second, due to data limitations, we are not able to examine the educational outcomes of older adults when virtually all of the educational transitions are complete. Finally and relatedly, the Add Health sampled respondents across some age span. Therefore, some respondents who were older when Wave 4 was administered had a better chance of completing their college degrees than did those who were much younger at the time of data collection. Despite these limitations, the results of the present study are helpful in understanding what socio-demographic and school-level factors and behaviors such as friendship preferences contribute to academic success.

## 6. Conclusions

The ongoing obesity epidemic presents a unique challenge for the US health care system. Although the precipitous pace of the epidemic is certainly a public health issue, it also has larger social implications that go beyond physical health [58,59]. Only now is the scientific community as well as the general public beginning to realize how high the social costs of obesity epidemic are. As this study demonstrates, being overweight or obese as a child has implications for that child’s educational attainment as an adult. Childhood obesity is indeed an academic risk factor. Academic outcomes are of high importance to individuals and society as a whole, given the link between educational attainment and employment opportunities in young adulthood. Academic underperformance due to obesity adds to the latent costs of the obesity epidemic, which have the potential to become unaffordable unless childhood obesity prevention is taken seriously and acted upon responsibly.

## Figures and Tables

**Table 1:** Weighted means (or percentages) and standard deviations of study variables by overweight/obesity status. SES: socio-economic status.

		Overweight (n = 2056)	Obese (n = 1451)	Healthy Weight (n = 8584)
1	Dependent Variables			
2	High School or Less	25%	37%	17%
3	Some College	40%	35%	45%
4	Bachelor’s Degree or Higher	33%	28%	40%
5	Race/Ethnicity			
6	African-American	22%	26%	13%
7	Asian American	3%	1%	7%
8	Latino	16%	19%	11%
9	Non-Hispanic whites	59%	54%	69%
10	Immigrant Generation			
11	Generation 1	4%	2%	7%
12	Generation 2	10%	7%	11%
13	Generation 3	85%	85%	78%
14	Family Effects			
15	Parents’ Education	14.3	13.5	15.3
16	Parents’ Income	4.5	4.2	5.2
17	Two-Parent Household	53%	44%	60%
18	Other Controls			
19	Age	28.2	28.3	28.2
20	Gender (Male)	47%	46%	49%
21	Network Factor			
22	Overweight Saliency	0.41	0.46	0.37
23	School-Level Variables			
24	Average SES	2.6	2.4	3.1
25	Percentage of Minority Students	29%	32%	24%

Note: All variables are from Wave 1 except for age and the dependent variables, which are from Wave 4.

**Table 2:** Predictors of high school graduation: odds ratios and their standard errors (in parenthesis).

	Models					
	1	2	3	4	5	6
**Weight Status**						
Overweight	1.16 (0.30) **	1.14 (0.30) **	1.11 (0.31) ***	1.16 (0.31) **	1.18 (0.30) ***	1.13 (0.31) **
Obese ^a^	1.24 (0.27) ***	1.24 (0.26) ***	1.20 (0.26) ***	1.22 (0.37) ***	1.19 (0.28) ***	1.20 (0.27) ***
**Race/Ethnicity**						
African-American ^b^		1.15 (0.25) ***	1.18 (0.26) ***	1.16 (0.27) ***	1.16 (0.27) ***	1.12 (0.26) *
Asian American ^b^		0.83 (0.32) ***	0.90 (0.33)	0.94 (0.34)	0.97 (0.34)	0.96 (0.34)
Latino ^b^		1.21 (0.30) ***	1.17 (0.29) ***	1.16 (0.30) **	1.14 (0.30) **	1.15 (0.29) ***
**Immigrant Generational Status**						
Immigrant Generation 1 ^c^			0.78 (0.31) ***	0.83 (0.32) ***	0.82 (0.32) ***	0.82 (0.32) ***
Immigrant Generation 2 ^c^			0.88 (0.26) *	0.86 (0.26) **	0.96 (0.27)	0.94 (0.27)
**Family Effects and Other Controls**						
Parents’ Education				0.80 (0.26) ***	0.82 (0.27) ***	0.84 (0.27) ***
Parents’ Income				0.79 (0.23) ***	0.80 (0.23) ***	0.85 (0.24) ***
Two-Parent Household				0.86 (0.23) ***	0.85 (0.24) ***	0.90 (0.23) *
Age				0.91 (0.27)	0.90 (0.27)	0.93 (0.27)
Male ^d^				1.00 (0.25)	1.05 (0.24)	1.04 (0.24)
**Network Factor**						
Overweight Saliency					1.19 (0.36) ***	1.16 (0.35) **
**School-Level Factors**						
Percentage of Minority Students						1.13 (0.44) *
Average SES						0.77 (0.41) ***
**Model Comparison Test ^†^**		739 ***	384	285	430 ***	405 ***
**Models Compared**		1 and 2	2 and 3	4 and 3	5 and 4	6 and 5

* *p* < 0.05; ** *p* < 0.01; *** *p* < 0.001. Note: reference categories: ^a^ healthy weight; ^b^ non-Hispanic white; ^c^ generation 3; ^d^ female; ^†^ The test is analogous to the nested F-test for ordinary least squares (OLS) regression models. It is based on the difference between the deviance statistics (defined as a -2 ln likelihood function value at convergence) of the models contrasted. The model comparison test is not applicable for models that differ only in the number of level-2 factors or cross-level interactions.

**Table 3:** Predictors of having some college education: odds ratios and their standard errors (in parenthesis).

	Models					
	1	2	3	4	5	6
**Weight Status**						
Overweight	1.11 (0.33) *	1.09 (0.33)	1.08 (0.34)	1.09 (0.34)	1.09 (0.34)	1.06 (0.34)
Obese ^a^	0.83 (0.27) ***	0.87 (0.26) **	0.86 (0.26) ***	0.84 (0.27) ***	0.89 (0.26) *	0.88 (0.26) *
**Race/Ethnicity**						
African-American ^b^		0.87 (0.27) ***	0.85 (0.27) ***	0.91 (0.26)	0.88 (0.25) **	0.86 (0.25) ***
Asian American ^b^		1.08 (0.33)	1.01 (0.31)	1.00 (0.32)	1.05 (0.33)	1.03 (0.32)
Latino ^b^		0.85 (0.27) ***	0.91 (0.28)	0.88 (0.26) ***	0.89 (0.27) *	0.90 (0.27) *
**Immigrant Generational Status**						
Immigrant Generation 1 ^c^			1.18 (0.30) ***	1.13 (0.32) ***	1.14 (0.32) ***	1.15 (0.32) ***
Immigrant Generation 2 ^c^			0.95 (0.24)	1.03 (0.25)	1.06 (0.28)	1.05 (0.27)
**Family Effects and Other Controls**						
Parents’ Education				1.18 (0.25) ***	1.17 (0.26) ***	1.14 (0.26) ***
Parents’ Income				1.34 (0.24) ***	1.31 (0.23) ***	1.27 (0.24) ***
Two-Parent Household				1.09 (0.23)	1.08 (0.24)	1.06 (0.24)
Age				1.06 (0.27)	1.04 (0.28)	1.05 (0.27)
Male ^d^				0.92 (0.23)	0.93 (0.23)	0.95 (0.23)
**Network Factor**						
Overweight Saliency					0.92 (0.33)	0.94 (0.32)
**School-Level Factors**						
Percentage of Minority Students						0.89 (0.45)
Average SES						1.17 (0.43) *
**Model Comparison Test ^†^**		688 ***	369 ***	403 ***	196	213
**Models Compared**		1 and 2	2 and 3	4 and 3	5 and 4	6 and 5

* *p* < 0.05; ** *p* < 0.01; *** *p* < 0.001. Note: Reference Categories: ^a^ healthy weight; ^b^ non-Hispanic white; ^c^ generation 3; ^d^ female; ^†^ The test is analogous to the nested F-test for OLS regression models. It is based on the difference between the deviance statistics (defined as -2 ln likelihood function value at convergence) of the models contrasted. The model comparison test is not applicable for models that differ only in the number of level-2 factors or cross-level interactions.

**Table 4:** Predictors of having a Bachelor’s degree or higher: odds ratios and their standard errors (in parenthesis).

	Models					
	1	2	3	4	5	6
**Weight Status**						
Overweight	0.86 (0.30) ***	0.84 (0.33) ***	0.82 (0.33) ***	1.09 (0.34)	1.09 (0.34)	0.87 (0.34) **
Obese ^a^	0.79 (0.26) ***	0.85 (0.25) ***	0.83 (0.26) ***	0.80 (0.25) ***	0.82 (0.25) ***	0.82 (0.26) ***
**Race/Ethnicity**						
African-American ^b^		0.77 (0.24) ***	0.76 (0.24) ***	0.81 (0.24) ***	0.83 (0.25) **	0.86 (0.25) ***
Asian American ^b^		1.21 (0.30) ***	1.12 (0.29) *	1.13 (0.30) **	1.08 (0.30)	1.02 (0.31)
Latino ^b^		0.82 (0.25) ***	0.84 (0.26) ***	0.83 (0.25) ***	0.85 (0.25) ***	0.88 (0.26) **
**Immigrant Generational Status**						
Immigrant Generation 1 ^c^			1.28 (0.30) ***	1.25 (0.32) ***	1.24 (0.32) ***	1.20 (0.32) ***
Immigrant Generation 2 ^c^			1.16 (0.24) ***	1.13 (0.25) *	1.09 (0.28)	1.06 (0.27)
**Family Effects and Other Controls**						
Parents’ Education				1.37 (0.24) ***	1.36 (0.24) ***	1.31 (0.24) ***
Parents’ Income				1.29(0.24) ***	1.26 (0.23) ***	1.22 (0.24) ***
Two-Parent Household				1.23 (0.23) ***	1.19 (0.23) ***	1.11 (0.24) *
Age				1.12 (0.29) *	1.10 (0.28)	1.08 (0.28)
Male ^d^				0.95 (0.21)	0.97 (0.21)	0.97 (0.23)
**Network Factor**						
Overweight Saliency					0.81 (0.36) ***	0.85 (0.37) **
**School-Level Factors**						
Percentage of Minority Students						0.79 (0.45) ***
Average SES						1.35 (0.43) ***
**Model Comparison Test ^†^**		771 ***	415 ***	458 ***	323 ***	427 ***
**Models Compared**		1 and 2	2 and 3	4 and 3	5 and 4	6 and 5

* *p* < 0.05; ** *p* < 0.01; *** *p* < 0.001. Note: Reference Categories: ^a^ healthy weight; ^b^ non-Hispanic white; ^c^ generation 3; ^d^ female; ^†^ The test is analogous to the nested F-test for OLS regression models. It is based on the difference between the deviance statistics (defined as -2 ln likelihood function value at convergence) of the models contrasted. The model comparison test is not applicable for models that differ only in the number of level-2 factors or cross-level interactions.
